# Do plants have a segregated germline?

**DOI:** 10.1371/journal.pbio.2005439

**Published:** 2018-05-16

**Authors:** Robert Lanfear

**Affiliations:** 1 Ecology and Evolution, Research School of Biology, Australian National University, Canberra, Australia; 2 Biological Sciences, Macquarie University, Sydney, Australia

## Abstract

For the last 100 years, it has been uncontroversial to state that the plant germline is set aside late in development, but there is surprisingly little evidence to support this view. In contrast, much evolutionary theory and several recent empirical studies seem to suggest the opposite—that the germlines of some and perhaps most plants may be set aside early in development. But is this really the case? How much does it matter? How can we reconcile the new evidence with existing knowledge of plant development? And is there a way to reliably establish the timing of germline segregation in both model and nonmodel plants? Answering these questions is vital to understanding one of the most fundamental aspects of plant development and evolution.

## Why care?

The germline is the immortal cell lineage that transmits the genome between generations. Understanding the segregation of germline and soma was a key step in our understanding of evolution [1] because once the germline is segregated, mutations that occur in somatic tissues cannot be inherited. It is typically understood that germline segregation occurs early in the development of most animals and late in the development of most plants [2–7]. Indeed, late germline segregation in plants is so widely accepted [6–26] that it is common to read that plants do not have a germline at all [3,27–44] (see also S1 Data, which contains full quotes in context). While this latter statement is probably not meant to be taken literally, its prevalence illustrates that the timing of germline segregation in plants is usually assumed to be a solved problem.

In this essay, I argue that the timing of germline segregation in plants is far from solved. A number of recent studies have suggested that some, and possibly most, plants possess an early-segregating and slowly dividing germline cell lineage that bears a striking resemblance to the animal germline [12,23,45]. These studies run counter to the prevailing wisdom that the plant germline is well understood and suggest instead that there is considerable uncertainty about its true nature. I start by reviewing the potential selective advantages of both early and late germline segregation in plants and outlining the differences between segregation and differentiation. I then review both the old and the new empirical evidence that bears on the nature of the plant germline. I argue that we cannot say with confidence whether the germline segregates early or late in plant development or whether the timing of germline segregation is conserved or varies among species. Nevertheless, I argue that there is strong empirical and theoretical evidence for a slowly dividing ‘functional germline’ in many plants, and that a functional germline might fulfill many of the same functions as the early-segregating germline in animals. I finish by suggesting approaches that could reveal the nature of the germline in a wide range of model and nonmodel plants. These approaches should help to resolve a range of open questions about the fundamental nature of the plant germline.

## What is germline segregation?

Germline segregation is the physical separation of the germline from the somatic cell lineages. Segregation is important because once the germline cell lineage is segregated from the somatic cell lineages, mutations occurring in somatic cells cannot be inherited. It was this observation that formed the basis of Weismann’s famous germ/soma distinction [1].

**Fig 1:**
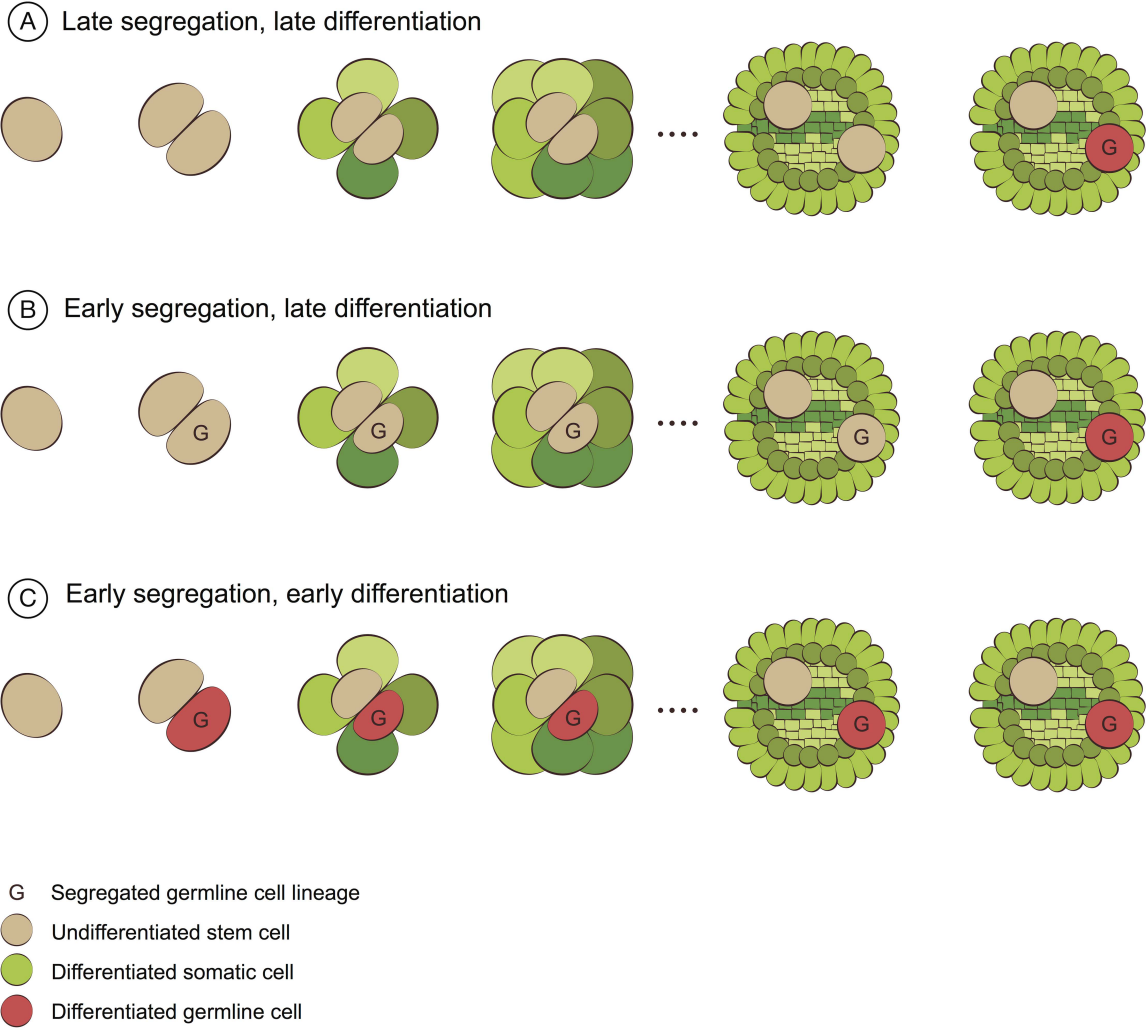
Three models of germline segregation. Cell lineages can be physically separated from each other whether or not they are differentiated. Because of this, germline segregation could occur earlier in development than germline differentiation. In this figure, once germline segregation occurs (marked by the appearance of the first cell with a ‘G’), additional germline cells (also marked with a ‘G’) are derived exclusively from existing segregated germline cells. I.e., the appearance of the first segregated germline cell (‘G’) denotes the timing of germline segregation. **(A)** Late germline segregation (cell marked with a ‘G’) co-occurs with late germline differentiation (cell marked in red), as is typically thought to be the case in plants. **(B)** Germline segregation (cell marked with a ‘G’) occurs early in development, and the cell lineage remains segregated until differentiation (cell marked in red) occurs late in development, a model which is not typically considered in plants or animals. **(C)** Early germline segregation (cell marked with a ‘G’) co-occurs with early germline segregation (cell marked in red), as is typically thought to be the case in animals. *Figure is adapted from [18] and created with the assistance of Ella Maru Studio.*

The developmental timing of germline segregation varies between species [7]. At one extreme, the germline cell lineage can segregate very late, such that it is not separated from the somatic cell lineages for most of development (e.g., Fig 1A). In this case, it is possible for somatic mutations to be incorporated into the germline cell lineage throughout most of development and subsequently passed on to future generations. At the other extreme, the germline cell lineage can segregate very early (e.g., at the two-cell stage), such that it remains separate from the somatic cell lineages for most of development (e.g., Fig 1C), which all but excludes the possibility for somatic mutations to be inherited. These extremes are two ends of a continuum: In principle, the germline could segregate from the somatic cell lineages at any point during development. In addition, the timing of germline segregation may vary between and within individuals of the same species. For example, in species without deterministic development there may be variation in the timing of germline segregation between individuals, and in organisms that produce multiple reproductive organs (such as many plants) there may be variation in the timing of germline segregation among those organs. In light of this, it is useful to think about the timing of germline segregation as a distribution that we should aim to characterize for a particular species. There is very good evidence that germline segregation occurs uniformly early in the development of many animals [7]. It is also widely accepted that germline segregation occurs uniformly late in the development of most plants (e.g., see S1 Data), but there is far less empirical evidence to support this view.

## Why does the timing of germline segregation matter?

Knowing the developmental timing of germline segregation is important for understanding species’ biology. For example, the timing of germline segregation can be a key determinant of the rate and spectrum of heritable mutation [6] and the rate of senescence [28] and has also been suggested to explain a variety of correlations between molecular evolution and life history [15].

The presumed differences between plants and animal germlines have led to speculation that the two groups might evolve in fundamentally different ways (e.g., [2,6]). But, if plants and animals share an early-segregating germline, then key aspects of their evolution may be more similar than is often thought. For example, the assumption that the plant germline segregates late in development has led to suggestions that plants should suffer from a high within-individual genetic load and may experience within-individual selection as they grow [46–49]. But, if the germline segregates early in development, then these ideas may need to be revisited and potentially revised.

Although an early-segregating germline would make plants and animals more similar in some respects, it could also amplify some of their differences. For example, it has been suggested that somatic mutations may contribute to plant fitness by creating variation that is useful for herbivore defence and plant immunity [4,50]. In both cases, the proposed fitness advantages stem from maintaining high within-individual variation for certain traits (chemotypes and immune genes, respectively), which could in principle be generated by a high somatic mutation rate. On the one hand, a high somatic mutation rate is more plausible in plants than in many animals because plant cells do not move, so plants cannot develop metastasising cancers [51]. But, if plants have a late-segregating germline, then a high somatic mutation rate also entails a high germline mutation rate, which is usually thought to be an evolutionary disadvantage [52,53]. An early-segregating germline could solve this problem by allowing natural selection to act independently on the somatic and germline mutation rates, leading to the evolution of a high somatic mutation rate (which may be advantageous in creating adaptive within-individual variation) and a low germline mutation rate (which may be advantageous in limiting the accumulation of new deleterious mutations in offspring). Regardless, it is clear that it is important to know the timing of germline segregation in order to have a complete understanding of plant life history and evolution.

## What is the difference between germline differentiation and germline segregation?

Differentiation and segregation are very different processes: Differentiation is a process of specialisation that occurs within a single cell, whereas segregation involves the physical isolation of one cell lineage from other cell lineages. During germline differentiation, a cell undergoes a recognisable change in gene expression which indicates that it has transitioned from a stem cell (which can develop into many different cell types) into a more specialised germline cell [13]. The timing of germline differentiation is relatively well understood in both animals and plants: The germline differentiates early in the development of most animals [7]—e.g., after a few cell divisions in mammals—but late in the development of most plants—e.g., just before flower formation in angiosperms [8]. Germline segregation is the physical isolation of the germline cell lineage from the somatic cell lineages, such that mutations that accumulate in somatic cell lineages cannot be transferred into the germline.

The causal relationship between differentiation and segregation is asymmetric: Differentiation causes segregation, but the reverse is not necessarily true. If differentiated cell lineages do not usually revert to stem cells, germline differentiation will cause simultaneous segregation of the germline cell lineage (e.g., Fig 1A and 1C). However, a cell lineage can be segregated from other cell lineages before it has differentiated (e.g., Fig 1B). Indeed, segregation before differentiation may be more likely to occur in plants than in animals because cell movement in plants is highly restricted [54]. This is important because it highlights that even though the germline cell lineage differentiates late in plant development [8], it could be segregated from the somatic cell lineages much earlier (e.g., Fig 1B). In other words, knowing the developmental timing of germline differentiation simply puts an upper bound on the developmental timing of germline segregation. And, since the germline differentiates late in the development of most plants, this leaves open the possibility that germline segregation could occur at any earlier point in development.

## What does theory tell us to expect?

Early-segregating germlines should provide an evolutionary advantage to multicellular organisms by reducing the accumulation of deleterious mutations in each generation. We expect most species to evolve per-generation mutation rates that are as low as possible given certain biochemical and population-genetic constraints, because most new mutations are deleterious [52,53,55,56]. Early-segregating germlines could help reduce per-generation mutation rates in at least three ways. First, germline segregation, by definition, blocks the transfer of somatic mutations into the germline cell lineage. This is important because we expect many somatic cells to have higher mutation rates than germline cells as a byproduct of the biochemical work that they do, so avoiding the inheritance of genetic material from somatic cells should reduce the per-generation mutation rate. Second, germline segregation would allow for the evolution of lower rates of cell division in the germline cell lineage, which is possible in the absence of germline differentiation because rates of cell division in plants can be controlled via external cell signals [54,57]. Importantly, a lower rate of cell division reduces the accumulation of mutations from both DNA copy errors and DNA damage [58], thus potentially providing a large reduction in the per-generation mutation rate. Reduced rates of cell division in the germline cell lineage could also be achieved without germline segregation, but if the germline is unsegregated, then even the occasional transfer of cells that had experienced a higher rate of cell division would increase the germline mutation rate. Third, segregation could facilitate selection to favour positioning the germline out of the way of mutagens such as UV radiation and heat, further reducing the per-generation mutation rate. This could also be achieved without germline segregation, but its effectiveness would be reduced if cells that been exposed to higher rates of DNA damage were occasionally transferred into the germline cell lineage.

Counter to the arguments proposed above, a recent study argued that late-segregating germlines could confer evolutionary advantages to certain organisms, potentially including plants [18]. The study develops a mathematical model of selection against deleterious mutation accumulation in the mitochondrial genome. It makes explicit the tension between two ways of reducing mutation accumulation in organellar genomes; on the one hand, an early-segregating and slowly dividing germline cell lineage can reduce mutation accumulation by reducing copying errors, as discussed above; but on the other hand, a late-segregating and rapidly dividing germline cell lineage can reduce mutation accumulation by increasing the segregational variance of organelles in the offspring (this works because there is more than one copy of each organelle in every cell [18]). The model suggests that early-segregating germlines should be favoured in organisms whose organellar genomes accumulate many mutations per generation and in which these mutations are derived primarily by DNA copying errors rather than background mutations. The authors suggest that these conditions hold for the mitochondrial genome of most animals, potentially explaining why it is common for animals to possess early-segregating and slowly dividing germlines. In contrast, the authors suggest that plant mitochondria display patterns of mutation consistent with a low ratio of copy errors to background mutations and conclude that their model explains why plants ‘do not have a germline’ [18]. Some aspects of plant molecular evolution suggest that this conclusion may be premature, despite the fact that the new model is clearly an important contribution. First, although plant mitochondria have a low point mutation rate, they have a surprisingly high structural mutation rate. For example, animal mitochondria almost always possess a single circular genome of 15–17 kb, but plant mitochondrial genomes vary by two orders of magnitude in both size (from 200 kb to more than 11 Mb) and structure (from 1 to 128 chromosomes) [59]. Thus, structural errors could form an important but overlooked component of deleterious mutation accumulation in plant mitochondria. Second, plants possess a second organellar genome, the chloroplast genome, which has a much higher mutation rate than the plant mitochondrial genome [60]. As with the structural mutations in mitochondrial genomes, chloroplast genome mutations will contribute an additional component of the total number of deleterious mutations in plants that was overlooked in the original model, potentially pushing plants into the category of organisms that the model predicts should evolve a slowly dividing and early-segregating germline [18].

## What does the empirical evidence suggest?

At least four lines of evidence have contributed to the acceptance of the late-segregating germline hypothesis. First, it is commonly assumed that the late-differentiation of plant germlines dictates their late segregation, although this is not necessarily the case (Fig 1 and section “What is the difference between germline differentiation and germline segregation?”). Second, it has been suggested that plants cannot have an early-segregating germline, because they can potentially develop flowers from any tissue [8]. However, this observation does not preclude the existence of an early-segregating germline: Normal development in plants could involve an early-segregating germline cell lineage which is simply regenerated from existing tissue if it is lost (e.g., due to herbivory) [61]. In this sense, if a flower develops from somatic tissue, the germline of that particular flower will have segregated late in development, while the germline of other flowers in the same individual plant could have segregated much earlier. Third, it is well known that cells in the layer of stem cells responsible for producing reproductive tissue in angiosperms (known as the L2 layer) can be displaced and/or replaced by unusual cell divisions from neighbouring cell layers [62,63]. But, while such occurrences could reset the timing of germline segregation when they occur, they do not preclude that normal development in plants could involve an early-segregating germline. Rather, both this and the potential for flowers to develop from any tissue highlight that the germline of different reproductive organs in the same individual may have segregated at different times, and thus that it is important to consider the distribution of germline segregation times in organisms with multiple reproductive organs. Finally, proponents of the early-segregation hypothesis lost the argument about the timing of germline segregation once before. In the mid-20th century, a group of French biologists suggested that the shoot apical meristem (SAM) contained a group of ‘waiting’ stem cells, the ‘meristem d’attente’, that showed a complete absence of mitotic division until the formation of the reproductive organs (reviewed in [64]). The existence of the meristem d’attente was never shown beyond doubt and was largely rejected on the basis of studies that showed that cells in the SAM were not completely quiescent [64] and analyses of chimeric plants that showed that the early embryo did not contain any cells that produced exclusively reproductive tissue [65–67]. However, while these observations do serve to reject the strict interpretation of the ‘meristem d’attente’ hypothesis, neither are sufficient to determine that germline segregation occurs late in development. A segregated germline need not be completely quiescent, and although the studies of chimeric plants show that germline segregation must occur after embryogenesis in a handful of small and short-lived model species, they leave open the possibility that germline segregation could occur at any point between the end of embryogenesis and the onset of flowering in these species, and they say relatively little about the timing of germline segregation in other plants, particularly those that are large and long lived.

One possibility consistent with the existing empirical evidence is that plants contain a ‘functional germline’ defined as a slowly dividing but unsegregated cell lineage that gives rise to both reproductive and somatic tissue [62]. This idea was initially developed from observations that most plants have a slowly dividing group of stem cells near the middle of the SAM, in a region often called the central zone (CZ; although, notably, this excludes plants such as ferns, which have just a single stem cell in the SAM). It has long been suspected that the slowly dividing cells in the CZ are those from which the differentiated germline is derived, and various authors have noted that reduced cell division in the CZ could reduce the accumulation of heritable mutations as plants grow [12,23,48,62,68]. Consistent with this, the cells in the CZ divide more slowly in trees than in smaller plants [62], just as one would predict if larger plants were under stronger selection to counter the accumulation of deleterious somatic mutations, and potentially explaining the observation that taller plants have slower rates of molecular evolution [15]. Furthermore, if the patterns of cell division in the CZ were organized hierarchically, it may be possible for the most slowly dividing cells in an unsegregated functional germline to divide remarkably few times during the lifetime of even large and long-lived plants [69]. Indeed, it was suggested some time ago that low rates of mitosis of cells in the CZ could mean that the germline could experience as few as 100 cell divisions per generation, even in long-lived trees [62]. If this were the case, a functional germline may confer almost all of the same benefits as an early-segregating germline. In other words, as long as the number of cell divisions per generation in the germline cell lineage (sometimes called the ‘cell depth’) is low, the timing of germline segregation may not matter very much.

Recent empirical studies put the existence of a functional germline in plants beyond doubt, and provide tantalizing evidence for an early-segregating germline in at least one species. In one study, researchers treated the cells of the *Arabidopsis* SAM with a dye that becomes visible only after cells have undergone mitosis [23]. Most cells in the SAM showed behaviour consistent with frequent and ongoing cell division—they stained brightly shortly after the dye was introduced (reflecting a short waiting period until the first cell division) and then progressively less brightly as time passed (reflecting the dilution of the stain from mother to daughter cells). Strikingly though, a small number of cells showed a very different pattern: They stained brightly and for the first time much later in development, just after the transition to flowering. One explanation for this is that these cells had remained quiescent for most of development, dividing for the first time around the transition to flowering [23]. If confirmed, these observations would suggest the presence of a quiescent (and thus, by definition, segregated) germline cell lineage in *Arabidopsis*, much as suggested by the ‘meristem d’attente’ hypothesis. This and another study have also shown that there are surprisingly few cell divisions per generation in two distantly related plants, *Arabidopsis* and tomato [45], providing very strong evidence for the existence of a functional germline in these species.

The recent observations in *Arabidopsis* and tomato have broad ramifications. The most recent common ancestor of *Arabidopsis* and tomato existed over 100 million years ago and defines a group (the core eudicots) that includes roughly two-thirds of all known flowering plant species [70]. Given the strong evidence for the existence of a group of slowly dividing cells in the SAM of many angiosperms [62] and the recent confirmation of a functional germline in *Arabidopsis* and tomato, it seems reasonable to suggest that most angiosperms probably have a slowly dividing functional germline. Intriguingly, this is at odds with the prediction that plants should be under selection to have a late-segregating and rapidly dividing germline cell lineage [18], perhaps indicating that the model on which this prediction is based does not apply to plants in the way that was initially proposed. Finally, if further experiments confirm that the germline of *Arabidopsis* and tomato segregate early in development, this would suggest that an early-segregating germline may be a common feature of angiosperm development.

In summary, many lines of empirical evidence point towards the existence of a slowly dividing functional germline in most angiosperms, but the timing of germline segregation remains unclear except for limited evidence for early germline segregation in *Arabidopsis*. Plant apical meristems differ substantially in their structure and organisation [62], and many of these differences should lead to differences in the nature of their germlines. For example, ferns and their allies possess a single stem cell in their apical meristem [62], precluding early germline segregation and limiting the extent to which a hierarchical pattern of cell division could limit the accumulation of somatic mutations [69]. Similarly, there are clear differences among monocots, dicots, and gymnosperms in the extent to which different cell populations in the apical meristem remain mutually isolated [62], suggesting that the distribution of germline segregation times may differ substantially between these clades. To make progress, we need to measure the distribution of germline segregation times in a broad range of plant species.

## How can we answer the question?

Model species will be vital for understanding the nature of the plant germline because of the range of methods that can be used to trace their cell lineages. Over short time periods, cell lineages can be observed directly with light or confocal microscopy (e.g., [71]). Over longer periods, cell lineages can be traced by pulse-labelling cells and following their descendants, by analysing sectors of tissue bearing naturally occurring or experimentally induced mutations, or by using reporter genes [23,65–67,72,73]. Recent advances show enormous promise for cell lineage tracing in model plants, because rather than live imaging (which is extremely difficult), they rely on cumulative genetic editing to concurrently label the developmental history of all cells within growing organisms [74,75]. Together, these approaches will allow for direct measurements of rates of cell division in the apical meristem, as well as clear delineation of the distribution of germline segregation times in model species. However, despite the various advantages of model species, there are relatively few of them, and they are all small and fast maturing. Theoretical considerations suggest that we might expect the benefits of an early-segregating germline to be amplified in larger and longer-lived plants because, all else being equal, we expect them to accumulate more mutations per generation than smaller and shorter-lived plants [3,6,62,69]. As such, a complete picture of the plant germline will require methods that can be applied to both model and nonmodel plants.

**Fig 2:**
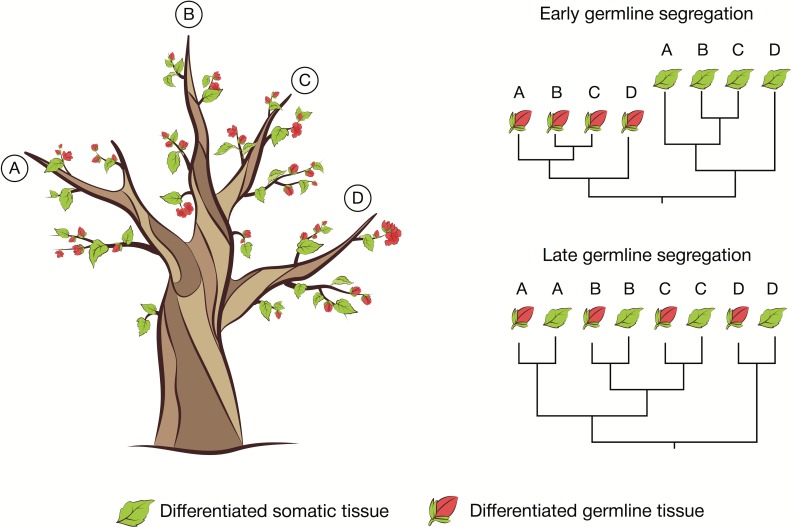
Cell lineage phylogenies can reveal the timing of germline segregation. Four paired samples (A-D) of somatic tissue (represented as a leaf) and germline tissue (represented as a flower) are taken from different branches of a single tree. Because plants accumulate somatic mutations as they grow, we expect a phylogeny of the 4 samples of either tissue to recapitulate the developmental histories of the cell lineages that led to those tissues. The phylogenetic relationships between somatic and germline tissues can reveal the timing of germline segregation. If the germline segregates early, then the germline tissues will group together on the tree (top phylogeny). If the germline segregates late (e.g., just before flowers form) then the germline and somatic tissue samples from each branch will group together in the tree. Intermediate timings of germline segregation will result in phylogenies with a structure intermediate between the two extremes shown here. Regardless, we expect the interrelationships among each of the 4 samples from a given tissue to recapitulate the physical structure of the tree, providing a useful positive control for difficult bioinformatics analyses. *Figure created with the assistance of Ella Maru Studio*.

New genome-sequencing technologies could allow us to determine the timing of germline segregation in almost any plant. It is now possible and affordable to detect naturally occurring somatic mutations that accumulate within individuals [20]. This approach could be leveraged to perform cell lineage tracing, particularly in plants that are large and long lived, without the need for experimental tools. Existing evidence suggests that although somatic and germline mutation rates are low, sufficient somatic mutations accumulate across the genome to make this approach feasible [20,76,77] (and even if this is not the case, it may be possible to artificially increase the mutation rate in some nonmodel species). The timing of germline segregation could be revealed by sequencing the genomes of paired somatic (e.g., leaf) and germline (e.g., pollen) tissues from multiple branches of a single plant and studying their interrelationships to reveal their underlying developmental history (Fig 2). If the germline cell lineage segregates late in development, then somatic and germline genomes from the same region of the plant will pair with each other on the cell lineage phylogeny, reflecting their developmentally recent common ancestry (Fig 2, bottom right panel). But, if the germline cell lineage segregates early in development—for example, before the first sampled branching event on a large tree—then a cell lineage phylogeny would show that the sampled germline tissues share a more recent common ancestor with each other than they do with any of the sampled somatic tissues (Fig 2, top right panel). More generally, the distribution of the timings of germline cell lineage segregation for any group of reproductive organs from a single individual can be revealed by the structure of a phylogeny of the tissues of that individual. The accuracy of this approach will depend on including the somatic tissue most closely related to the germline tissue in the cell lineage phylogeny (e.g. in plants that derive their germline from the L2 cell layer, somatic tissue that is also derived from this cell layer should be included in the phylogeny).

A cell lineage phylogeny for a single plant could also reveal differences in mutation rates between germline and somatic cell lineages. The branch lengths of cell lineage phylogenies reflect the product of the mutation probability per cell division and the number of cell divisions. If the germline cell lineage has a lower mutation rate than the somatic cell lineages, we would expect branches of the phylogeny that represent germline cell lineages to be shorter than their somatic counterparts (e.g., Fig 2). Existing statistical tests can reveal whether there are measurable differences between the overall mutation rates of the different cell lineages represented on a phylogeny [78], allowing us to test whether the mutation rate of the germline cell lineage is lower than that of the somatic cell lineages, as we might expect if the primary function of the germline is to preserve an accurate copy of an individual’s genome for future generations.

## Conclusion

I have argued in this essay that, contrary to the widely accepted view, we know relatively little about the timing of germline segregation in plants. Different theoretical considerations predict different outcomes, and it is perhaps too early to say which theory is most likely to hold for plants. The empirical evidence suggests that, at least for some model plants, germline segregation must occur after embryogenesis but before the transition to flowering, but this leaves open that germline segregation could occur at almost any point during development. There is strong evidence for a slowly dividing functional germline in plants, which I argue could confer many of the same benefits as an early-segregating germline. These arguments suggest that it is misleading to claim that plants lack a germline and that it may be premature to suggest that plants and animals evolve in fundamentally different ways based solely on the properties of their germlines. I argue that because plant development is plastic, we should aim from the outset to estimate the distribution of germline segregation times typical for a species, and I suggest a method that might be used to measure that distribution in model and nonmodel plants.

The timing of germline segregation is known to vary widely among animals [7], and we should not expect plants to be any different. Indeed, given the fundamental differences between the SAMs and the life history strategies of major clades of plants [62], perhaps we should expect from the outset that the timing of germline segregation will vary substantially among plant species. It is my hope that recent technological advances, and perhaps this essay, will help to spur research into this fascinating area.

## Supporting information

S1 Data A tab-separated values files (which can be opened in a plain text editor, Google Sheets, Microsoft Excel, or any spreadsheet editor) that contains quotes from 42 papers. There are five columns: (i) ‘Reference’ provides the full reference; (ii) ‘Category’ describes whether the quote was determined to suggest that plants do not have a germline at all or that the plant germline is set aside late in development; (iii) ‘Quote’ is the quote of interest; (iv) ‘Context’ gives the full context of the quote, usually the paragraph in which it appears; (v) ‘Page’ gives the page number on which the quote is found.
(TSV)

## Abbreviations

CZ: central zone
SAM: shoot apical meristem
